# Oxytocin treatment in pediatric populations

**DOI:** 10.3389/fnbeh.2014.00360

**Published:** 2014-10-16

**Authors:** Adrienne E. Taylor, Hsu-en Lee, Femke T. A. Buisman-Pijlman

**Affiliations:** ^1^Department of Psychological Medicine, Women's and Children's HospitalAdelaide, SA, Australia; ^2^School of Medicine, The University of AdelaideAdelaide, SA, Australia; ^3^Discipline of Pharmacology, Faculty of Health Sciences, School of Medical Sciences, The University of AdelaideAdelaide, SA, Australia

**Keywords:** oxytocin, neuropeptide, attachment, pediatric populations, intranasal oxytocin

## Abstract

The role of endogenous oxytocin as neuromodulator of birth, lactation and social behaviors is well-recognized. Moreover, the use of oxytocin as a facilitator of social and other behaviors is becoming more and more accepted. Many positive effects have been attributed to intranasal oxytocin administration in animals and humans; with current research highlighting encouraging advances in its potential for use in mental health disorders. The new frontier will be investigating the effective use of oxytocin in pediatric populations. Limited animal data is available on this. Large-scale human studies focusing on autism are currently under way, but many other possibilities seem to lie in the future. However, we need to know more about the risks and effects of repeated use on the developing brain and body. This paper will provide an overview of the current understanding of the role of endogenous oxytocin and its related neuropeptide systems in influencing behaviors, in particular attachment, and will review (a) the literature on the use of intranasal oxytocin in young animals, children (age range birth-12 years) and adolescents (age range 13–19 years), (b) the expected benefits and risks based on the current research, and (c) the risks of oxytocin in children with severe psychopathology and early life trauma. The paper will conclude with a clinical perspective on these findings.

## Introduction

Current understanding of oxytocin and its related neuropeptide systems is growing at an exponential rate. Oxytocin's centrally acting role in behavioral modulation and attachment is increasingly recognized (Feldman, 2012; Churchland and Winkielman, 2012; Tops et al., 2014). Recent studies (Broadbear et al., 2014; Love, 2014; Tops et al., 2014) have been able to highlight a neurobiological model for these behavioral changes, and outline with greater specificity the complex interaction between oxytocin and other neuropeptides and monoamines. These findings have been applied and expanded to the field of addiction, and behavioral neuroscience (Kovacs et al., 1998; Rilling et al., 2012; Buisman-Pijlman et al., 2014b; Williams and Johns, 2014; Zanos et al., 2014). Many positive effects have now been attributed to intranasal oxytocin in the adult population (Veening and Olivier, 2013), however there are multiple questions that remain unanswered in regards to the safe and effective use of intranasal oxytocin in the child and adolescent population. In particular, there is a lack of evidence in regards to the effects of both acute and chronic exposure on the developing brain. This paper aims to provide a review of current knowledge in relation to this area, and highlight both theoretical and clinical gaps in research evidence and data regarding what will likely be an increasing area of interest in the management of common childhood and adolescent psychiatric disorders in future.

This paper will briefly review the current understanding of the role of endogenous oxytocin and its related neuropeptide systems in influencing behaviors, in particular attachment, and will review (a) the literature on the use of intranasal oxytocin in young animals, infants, children and adolescents, (b) the expected benefits and risks based on the current research, and (c) the risks of oxytocin in children with severe psychopathology and early life trauma. Finally this paper aims to utilize the current knowledge base to assist in providing a framework for the direction of potential further clinical application and will provide examples for future research questions.

## Oxytocin and its related neuropeptide systems

Oxytocin, a hormone synthesized by the hypothalamus and subsequently secreted by the posterior pituitary gland, is a neuropeptide well-known for its neuro-modulatory role in lactation, child-birth, mother-infant bonding (Love, 2014), and more recently for its role in social affiliation and partner attachment in adults (Buisman-Pijlman et al., 2014b; Tops et al., 2014). Oxytocin's centrally acting effects form the basis of research stemming from Nobel laureate in Chemistry Vincent du Vigneaud's early descriptions and syntheses of oxytocin and vasopressin in 1953 (Love, 2014; Sarnyai and Kovacs, 2014). Oxytocin has it's effect through the Oxytocin receptor and Vasopressin 1A Receptor. Immunohistochemical studies have revealed that magnocellular oxytocin neurons of paraventricular, supraoptic, and accessory hypothalamic nuclei via local dendritic and distant axonal release of oxytocin modulate various forms of behaviors (Lee et al., 2009; Knobloch et al., 2012; Dolen et al., 2013). It is possible that axonal projections of oxytocin neurons can be altered in patients afflicted with neurodevelopmental diseases, which may impair oxytocin signaling and contribute to defects in social behavior. Such pathophysiological mechanisms require further research.

Oxytocin has been shown to have a relationship with both vasopressin and serotonergic neural pathways (Broadbear et al., 2014), with increasing evidence suggesting that oxytocin and vasopressin's individual and combined modulatory influences on related neurotransmitter systems, including dopaminergic, serotonergic, and endogenous opioid system play a crucial role in the brain's motivational and reward pathways (Love, 2014), and behaviors related to stress-responses and addiction (Buisman-Pijlman et al., 2014b; Tops et al., 2014). Oxytocin has also been implicated in modulating the reactivity of the hypothalamic-pituitary-adrenal axis (Buisman-Pijlman et al., 2014b), thus in part determining the extent of psychological and physiological response to stress (Feldman, 2012).

This expanding knowledge base has underpinned adult human and animal studies administrating intranasal oxytocin as a modulator of behaviors, for example, those associated with substance abuse (McGregor and Bowen, 2012; Pedersen et al., 2013). Authors have outlined the rationale for intranasal administration of oxytocin in research, based on this administration route bypassing the blood-brain barrier, and increasing the half-life of the delivered substance in cerebrospinal fluid thus ensuring maximization of centrally acting effects (Veening and Olivier, 2013). Intranasal delivery accesses rostral and possibly other brain areas following absorption through the nasal epithelium (Veening and Olivier, 2013). In the pediatric population, intranasal delivery of oxytocin is likely to be the easiest, least intrusive and most preferable mode of administration given pragmatic factors relating to level of insight into treatment, and aversion behaviors, however this needs further investigation.

From a clinical perspective, Intellectual Disabilities, Autism Spectrum Disorders (ASDs) and ADHD are common neurodevelopmental disorders presenting in childhood. Whilst neurodevelopmental disorders frequently require ongoing treatment and management in adolescent populations, this is an age group in which affective disorders, anxiety disorders and eating disorders, or traits thereof, also start to manifest and may require clinical intervention. In the child and adolescent this cohort, disorders are frequently and universally, however not exclusively, underpinned by a developmental history characterized by attachment difficulties or early life trauma.

## Oxytocin's role in attachment

An important precursor to the investigation of oxytocin's role within pediatric populations is the concept of attachment. From a theoretical perspective, Bowlby defines attachment as “*a biological instinct…Attachment behavior anticipates a response by the attachment figure which will remove threat or discomfort*” (Bowlby, 1960). Oxytocin has been implicated in priming the bond between a mother and child (Love, 2014), and it is known from animal studies that the development of the oxytocin system continues throughout the postnatal period in an experience-dependent manner (Oreland et al., 2010). Given that development of secure attachment is in part reliant on an infant's salience toward their primary caregiver, evidence that administration of oxytocin improves the salience of social cues further highlights the importance of oxytocin in the process of attachment (Love, 2014; Tops et al., 2014).

## Variations in the oxytocin system

Notably, findings from animal and human studies display that there are individual variations within basal oxytocin levels and in reactivity of the system. Whilst genetics contribute in part, environmental influences such as social experiences, stress and trauma early in life have also been shown to affect this variation. Buisman-Pijlman et al. (2014b) outlined that these influences can induce long-term changes to the oxytocin system, having a subsequent impact on an individual's susceptibility to addiction and relapse. Feldman (2012) also noted that the human oxytocin system is sensitive to both positive early social experiences and negative experiences such as separation. Rather than a static feedback system, it is now more accepted through recent studies that the human oxytocin system functions in a complex context-dependent manner (Feldman, 2012; Guastella and Macleod, 2012). Factors aside from genetic influence that have been implicated in variations in an individual's oxytocin system include prenatal maternal stress and substance use (Johns et al., 1998), early life trauma including parental separation (Opacka-Juffry and Mohiyeddini, 2012), and childhood sexual abuse (Heim et al., 2009). A sound understanding of these factors will be of importance to assess the risks and benefits of exogenous oxytocin in the pediatric population.

## Current evidence on the use of intranasal oxytocin in young animals, infants, children, and adolescents

In the adult population, literature on the use of intranasal oxytocin has spanned the last six decades in research, with early reports focusing on its role in obstetrics (Hendricks and Pose, 1961; Cohen et al., 1962), followed by exploration of oxytocin's possible application to the field of psychiatry, in particular, obsessive compulsive disorder (Ansseau et al., 1987; Den Boer and Westenberg, 1992) and symptoms of post-traumatic stress disorder (Pitman et al., 1993; Olff, 2012).

In the last 10 years, authors have highlighted oxytocin's prosocial effects in the adult population, categorized further into “trust” (Baumgartner et al., 2008), “mind-reading” and “face-processing and memory” (Heinrichs et al., 2003; Beetz et al., 2012) and stress-response in animals (Parker et al., 2005). It is these benefits, in addition to genetic studies displaying a consistent pattern of oxytocin involvement in ASDs (Campbell et al., 2011), that have led to an interest in the promise of treatment of ASDs with intranasal oxytocin. A recent systematic review of randomized control trials of oxytocin intervention in autism by Preti et al., noted that whilst promising findings in the measures of emotion recognition and eye gaze, there is need for better powered studies prior to valid clinical recommendations being made (Preti et al., 2014).

Prior to recent years, animal studies relating to the effects of oxytocin have mainly focused on single dose administration, and in an adult population. On review of current animal data (Table 1), there were four studies identified investigating administration of oxytocin via intraperitoneal injection, and three studies using intranasal oxytocin in young.

**Table 1:** **Acute and long-term effects of oxytocin administration in young animals**.

**Study**	**Study Type**	**Sample**	**Gender**	**Focus**	**Dose of OXT and route of administration**	**Duration of treatment**	**Behavioral Observations**
Bales and Perkeybile, 2012	Prairie voles	*N* = 89;	Male and female	Acute and long-term	IN:	21 days	“Prosocial effects” identified following single dose administration. Impaired formation of pair-bond in male voles in dose-dependent curve
	Blind study: OXT or saline;	EXPOSED PND 21–42 days old (adolescence)			Low = 0.08 IU/kg		
					Med = 0.8 IU/kg		
					High = 8.0 IU/kg		
Bales et al., 2007	Prairie voles; saline or one of four dosages of oxytocin	Neonates (PND1); *N* = 8–12 per group	Female	Long-term	2, 4 or 8 mg/kg injection	1 day	Effects differed per dose, but not linear. Both increase and decrease the propensity to display behaviors such as pair-bonding
Bales et al., 2004	Prairie voles; saline, oxytocin or antagonist	Neonates (PND1)	Male and female	Long-term	IP	1 day	Different effects on social and anxiety behavior in the sexes
Rault et al., 2013	Pigs	*N* = 24; EXPOSED PND 1–3; (neonates)	Male and female	Acute and long-term	IN 24 IU	3 days (daily dosing at 1,2,3 days of age)	Neonatally OT-administered pigs received increased aggressive interactions and showed aggressive behaviors, increased locomotion, less social contact and raised cortisol levels at 17 days and 8 weeks. Decreased responsiveness on dexamethasone suppression test at 11 weeks
	OXT or saline; acute and long-term						
Simpson et al., 2014	Newborn macaques	*N* = 28;	Male and female	Long-term	IN dose unquantified	7 days	Increased affiliation, communication gestures
	Blind study: OXT or saline	Newborn macaques					
	Long-term effects	EXPOSED PND 7-14					
Bowen et al., 2011	Wistar Rats	*N* = 48; PND 33–42 (early adolescence)	Male	Long-term	1 mg/kg IP	PND 33–42	Less anxiety, more sociability and reduced motivation to consume alcohol. Changes to OX system
Suraev et al., 2014	Long-Evans Rats, Oxytocin or selective	PND 28–55; (Adolescence)	Male	Acute and long-term effects	0.5–1 mg/kg, IP; intermittent regime of OT exposure (10 doses in total)	During entire adolescence PND 28–55	Acute decrease in social play and possible sedation;
	OXTR agonist						Long-term: increase social interaction;
							Increased plasma oxytocin.
							Selective oxytocin agonist could not fully mimic effects, implicating V1aR in some effects

*PND, postnatal day; IN, Intranasal; IP, Intraperitoneal*.

Studies focusing on intraperitoneal oxytocin injection reinforced inconsistent findings displayed in earlier data according to dose and measured outcome variable, and further highlighted that in the short-term, oxytocin administration may lead to a decrease in positive social behaviors (Suraev et al., 2014), whilst in the long term contribute to heightened social interaction and diminished display of anxiety (Bowen et al., 2011) (see Table 1).

All three animal studies investigating intranasal oxytocin involved repeated dosing. Firstly, Bales et al., found repeated dosing during adolescence had long-term effects on partner preference in prairie voles (Bales et al., 2013). Secondly, Rault et al., found that neonatal pigs displayed increased aggressive behaviors and locomotion, and diminished social contact following daily intranasal oxytocin administration in days 1–3 of life. In addition, there was decreased responsiveness on dexamethasone suppression test at 11 weeks, suggesting repeated intranasal oxytocin administration in early life can cause chronic dysregulation of the hypothalamic-pituitary axis (Rault et al., 2013). Most recently, Simpson et al., found that in newborn rhesus monkeys, administration of daily intranasal oxytocin increased an infant's affiliative communication gestures and decreased salivary cortisol levels (Simpson et al., 2014). In a similar trend identified in the administration of oxytocin via intraperitoneal injection in young animals, the use of intranasal oxytocin produced largely variable results, which appeared to be dose-dependent. Whilst prosocial effects and a decrease in salivary cortisol levels were some of the positive outcomes observed, authors also commented on intranasal oxytocin administration in young animals having the potential to increase aggressive behaviors, impair pair-bond formation, and lead to disruption of the HPA-axis.

A thorough review of current literature found that there were only six human studies (Table 2) focusing on the use of intranasal oxytocin in a child or adolescent population. Of these studies, five occurred in populations with a diagnosis of ASDs (Guastella et al., 2010; Gordon et al., 2013; Tachibana et al., 2013; Dadds et al., 2014; Guastella et al., 2014), and one in a population with Fragile X Syndrome (Hall et al., 2012). Of these six studies, Guastella et al.'s (2014) RCT investigating the effects of a course of intranasal oxytocin on social behaviors in youths with ASDs, and Tachibana et al., were the only groups to investigate chronic administration of intranasal oxytocin over 2, and 6 month periods, respectively, although there was notably a small sample size in the later study. Moreover, only three of the relevant studies found on review included children under the age of 12 (Gordon et al.; Dadds et al.; Tachibana et al.), with a clear lack of data pertaining to the effect of intranasal oxytocin administration in the neonatal and early childhood/pre-pubertal stages of development, a time during which the oxytocin system displays its highest degree of neuroplasticity. From a broader perspective, review of the current literature of intranasal oxytocin use in child and adolescent populations highlighted significant limitations to inferring clinically significant results, and recognizing consistent patterns of effect between studies, including: small sample sizes, limited data on chronic use compared to single-dose administration, and a predilection toward male subjects.

**Table 2:** **Intranasal oxytocin administration in children and adolescents (0–18 year old)**.

**Study**	**Study type**	**Sample**	**Gender**	**Primary targeted disorder**	**Dose of OXT (IU)**	**Duration of treatment**	**Side effects**	**Clinical outcome**
Dadds et al., 2014	Double-blind RCT: IN OXT vs. placebo	*n* = 38;	Male	Autism Spectrum disorders	12 or 24 dose calculated on weight	Daily dosing over 4 consecutive days	No significant side-effects reported	No significant improvement in emotional recognition, social interaction skills, or general behavioral adjustment on pre vs. post dose assessments
		Mean age: 11.2 ± 2.6 years						
		(range:7–16 years)						
Guastella et al., 2014	Double-blind, placebo-controlled trial	*n* = 50; Age range 12–18 years	Male	Autism Spectrum Disorders	18 or 24 IU twice daily dosing	Twice daily dosing for 8 weeks	No side effects reported	No overall significant improvement/ clinical efficacy identified in subjects receiving course of intranasal oxytocin
	IN OXT vs. placebo					Follow-up at baseline, a 4,8,12 weeks post treatment		
Guastella et al., 2010	Crossover design: IN OXT vs. placebo	*n* = 16;	Male	Autism Spectrum Disorders	Single dose 18–24 IU depending on age	Intervals between sessions unspecified	No significant side-effects	OXT improved performance on Reading the Mind in the Eyes test (RMET) in 60% subjects
		Mean age: 14.8 ± 2.4						
		(range:12–19)						
Tachibana et al., 2013	Single-armed open label study	*n* = 8;	Male	Autism Spectrum Disorders	Incremental doses every 2 months from 8 IU, 16 IU, 24 IU with 1–2 week placebo period prior to each dose increase	6 months active treatment, up to 6 weeks total placebo prior to each dose increase	No side effects reported	6 of 8 subjects displayed improved scores on the ADOS-G. Caregivers report improvement in reciprocal communication
	IN OXT vs. placebo	Age range 10–14 years						
Hall et al., 2012	Crossover design: IN OXT vs. placebo	*n* = 8;	Male	Fragile X Syndrome	24–48 IU	OXT vs. placebo challenges separated by 1 week	No side effects or adverse events reported	Improved eye gaze frequency in response to 24 IU IN OXT. Decrease in salivary cortisol levels in response to 48 IU OXT
		Mean age:21.3 ± 5.1 years						
		(range: 13–28)						
Gordon et al., 2013	Randomized, Double-blind, crossover functional MRI study	*n* = 21;	Female	High-functioning	7–11 years	Single dose	No significant side-effects reported	Increased activity in “social regions”/neural networks on fMRI imaging using the RMET test
	IN OXT vs. placebo	Age range 8–16.5 years	*n* = 3	Autism Spectrum Disorders	dose = 12 IU			
			Male		12–15 years			
			*n* = 18		dose = 18 or 24 IU			
					16–19 years			
					dose = 24 IU		

*IU, International Unit*.

Studies identifying beneficial clinical outcomes to intranasal oxytocin administration were (1) Gordon et al., who found improved performance in detecting socially salient information on a well-validated fMRI emotion judgment task (“Reading the Mind in the Eyes Test”) following intranasal oxytocin, in addition to identifying that oxytocin enhances activity in social brain regions (see Table 2) (Gordon et al., 2013), (2) Guastella et al., in their 2010 crossover study who displayed improved performance on the RMET in 60% of subjects following intranasal oxytocin administration (Guastella et al., 2010), (3) Hall et al., who reported improved frequency of eye gaze at a specific dose oxytocin, in addition to decreased salivary cortisol levels in subjects receiving a specific dose (Hall et al., 2012) and (4) Tachibana et al.,'s study revealing six of a total 8 male youths with a primary diagnosis of ASD displayed improved performance on communication and social interaction domains of the Autism Diagnostic Observation Schedule-Generic (ADOS-G) following incremental dose increases of intranasal oxytocin over a 6 month period (see Table 2).

The most recent studies by Dadds et al., and Guastella et al., do not report identified significant clinical benefit following the administration of oxytocin.

Despite an evolving scope of current animal and human research data relating to the use of intranasal oxytocin in adults, studies relevant to young animals, and the pediatric population remain limited to date. Factors contributing to this may be related the uncertainty of risks of single, or multiple oxytocin dose administrations on the developing brain, in addition to an absence of clear methods to measure outcome (Miller et al., 2013). Review highlighted no significant reporting of side effects or adverse events in the studied populations, with multiple studies referring to intranasal oxytocin being well-tolerated.

## The expected benefits and risks of intranasal oxytocin in pediatric populations

In assessing expected risks and benefits of oxytocin administration in the pediatric population, it will be important to first consider basic principles of neuroadaptation, including the development of tolerance, changes in receptor numbers, and receptor sensitivity, related to repeated exposure of any drug.

Based on current research data in adult animal and human populations, and the promising findings noted in behavioral modulation (Miller, 2013; Love, 2014), translational studies to the pediatric population could too hold promise. There is a potentially broad scope of application for the use of intranasal oxytocin based on mental health disorders or presentations that are characterized by a decreased responsiveness to social cues, emotional dysregulation and aggression. It would also be of interest to determine if the positive findings in the field of oxytocin and substance addiction (Bowen et al., 2011; Buisman-Pijlman et al., 2014a) were reproducible in younger populations.

As outlined by Miller et al. (2013) in their study reviewing the role of gender in regards to symptomatology in ASDs, there are variations in oxytocin and vasopressin identified between sexes (Miller et al., 2013). Broader application of this principle and an understanding of baseline variations in endogenous oxytocin levels between sexes will likely be important in future research involving administration of intranasal oxytocin to both young males and females for various mental health disorders.

In recent reviews, authors have commented on “cascading effects” of intranasal oxytocin (Veening and Olivier, 2013) whereby administration of intranasal oxytocin also stimulates release of peripherally acting oxytocin (Veening and Olivier, 2013). One rat study did report increased basal levels of oxytocin after long-term administration during adolescence (Suraev et al., 2014); the opposite could be possible as well-depending on the age of exposure. Potentially detrimental effects of inadvertently raising systemic levels of oxytocin remain unclear both in the adult and pediatric population, and would require further investigation from a safety perspective.

## Risks of oxytocin in children with severe psychopathology and early life trauma

As outlined, the oxytocin neuropeptide system in humans remains in an evolutionary state with ongoing plasticity throughout infancy, early childhood, and during adolescence (Buisman-Pijlman et al., 2014b).

Those impacted by significant early life trauma are more likely to display a heightened degree of emotional dysregulation (Antoniadis et al., 2012). It is well-recognized in psychiatric practice that emotional dysregulation, impulsivity, and relationship instability are clinical features of psychopathology in Borderline Personality Disorder that are likely to be in an evolutionary state during adolescence (Barnow et al., 2012). The role of oxytocin in modulating the relational capacities in children and adolescents has been considered extensively in the literature, particular in relation to attachment disorders, anxiety and autism-spectrum disorders (Meyer-Lindenberg et al., 2011; Frijling et al., 2014).

The effect of intranasal oxytocin on these subjects might be difficult to predict. In addition, there will need to be a heightened awareness of the interconnectivity of for example the dopaminergic, serotonergic, and noradrenergic systems (Tops et al., 2014), and potential effects this poses, with oxytocin administration. In a clinical subgroup already plagued by attachment and relational difficulties, further concern is warranted given the risk of oxytocin administration leading to long-lasting behavioral and social, and neuroendocrine consequences (Miller, 2013).

The current research in fact suggests that oxytocin administration (intranasally) in the clinical settings for autism, social anxiety disorder, post-natal depression, obsessive-compulsive disorder, schizophrenia, borderline personality disorder, and post-traumatic stress disorder, yield minimal improvement in emotion recognition and interpersonal trust. In the context of developmental trauma, positive effects (behaviorally or neurobiologically) were in fact lowered or absent (Bakermans-Kranenburg and van I Jzendoorn, 2013). It may be more feasible to identify improvements in disorders that have a homogenous etiology and little comorbidity.

## Discussion

An evolving wealth of animal and human data relating to the neuro-modulatory role of the endogenous oxytocin system, its interactions with other neural pathways, and its implications on behaviors from a neuroscience perspective is expanding at an exponential rate (Tops et al., 2014). There is evidence highlighting (1) the importance of oxytocin in attachment (Love, 2014) and (2)the factors that may lead to individual variations in the endogenous oxytocin system including early life trauma and environmental factors (Buisman-Pijlman et al., 2014b). This highlights further potential research questions based around whether attachment difficulties may be in part modifiable not only through psycho-education and implementation of behavioral strategies, but through implementation of pharmacotherapies for high-risk populations.

Furthermore the benefits of intranasal oxytocin administration in the adult population are now also becoming clearer, and the potential clinical applications more far-reaching. Researchers are now investigating the potential use of intranasal oxytocin in the treatment of depression as an adjunct to selective serotonin reuptake inhibitors (Emiliano et al., 2007), anxiety (Guastella et al., 2009), and more recently, anorexia nervosa (Kim et al., 2014) with promising preliminary results findings. This data would be of high clinical relevance if translated to trials targeting the adolescent population in which affective, anxiety and eating disorders are also prevalent. There are limited animal and human studies reflecting consistent results to date in pediatric populations, with the vast majority focusing on the use of intranasal oxytocin in modifying behaviors related to ASDs. There are fundamental considerations relating to neuroplasticity, neuro-adaptation and the impact of significant psychopathology that remain inadequately explored, and will likely inform further discussions around the safety of intranasal oxytocin as a therapeutic intervention. Based on our knowledge of the context-dependent nature (Guastella and Macleod, 2012), and plasticity of oxytocin system (Buisman-Pijlman et al., 2014b), animal and human studies are challenging to translate to pediatric populations. In addition, further questions relating to sensitive and specific methods of measuring outcomes in potential trials need consideration. Addressing these issues could lead to a broader wealth of research relating to oxytocin in pediatric populations, findings of which would likely continue to inform the way in which therapeutic interventions in the child and adolescent population are considered in the future.

### Conflict of interest statement

The authors declare that the research was conducted in the absence of any commercial or financial relationships that could be construed as a potential conflict of interest.
